# Measuring Device for Air Speed in Macroporous Media and Its Application Inside Apple Storage Bins

**DOI:** 10.3390/s18020576

**Published:** 2018-02-13

**Authors:** Martin Geyer, Ulrike Praeger, Ingo Truppel, Holger Scaar, Daniel A. Neuwald, Reiner Jedermann, Klaus Gottschalk

**Affiliations:** 1 Department Horticultural Engineering, Leibniz Institute for Agricultural Engineering and Bioeconomy (ATB), Max-Eyth-Allee 100, 14469 Potsdam, Germany; upraeger@atb-potsdam.de (U.P.); itruppel@atb-potsdam.de (I.T.); 2 Department Postharvest Technology, Leibniz Institute for Agricultural Engineering and Bioeconomy (ATB), Max-Eyth-Allee 100, 14469 Potsdam, Germany; hscaar@atb-potsdam.de (H.S.); kgottschalk@atb-potsdam.de (K.G.); 3 Competence Centre for Fruit Growing—Lake Constance, Ravensburg, Germany; University of Hohenheim, Institute of Crop Sciences, Section Crop Physiology of Specialty Crops, 70593 Stuttgart, Germany; Neuwald@kob-bavendorf.de; 4 Institute for Microsensors, -Actuators and -Systems (IMSAS), University Bremen, Otto-Hahn-Allee NW1, 28359 Bremen, Germany; rjedermann@imsas.uni-bremen.de

**Keywords:** air speed logger, air velocity, calorimetric principle, fruit, vegetable, storage, produce bulk, cold store

## Abstract

In cold storage facilities of fruit and vegetables, airflow is necessary for heat removal. The design of storage facilities influences the air speed in the surrounding of the product. Therefore, knowledge about airflow next to the product is important to plan the layout of cold stores adapted to the requirements of the products. A new sensing device (ASL, Air speed logger) is developed for omnidirectional measurement of air speed between fruit or vegetables inside storage bins or in bulk. It consists of four interconnected plastic spheres with 80 mm diameter each, adapted to the size of apple fruit. In the free space between the spheres, silicon diodes are fixed for the airflow measurement based on a calorimetric principle. Battery and data logger are mounted inside the spheres. The device is calibrated in a wind tunnel in a measuring range of 0–1.3 m/s. Air speed measurements in fruit bulks on laboratory scale and in an industrial fruit store show air speeds in gaps between fruit with high stability at different airflow levels. Several devices can be placed between stored products for determination of the air speed distribution inside bulks or bin stacks in a storage room.

## 1. Introduction

The knowledge of the spatial distribution of airflow in complex geometries is essential for optimization in several technical applications. Due to the increasing computational power with advanced workstations, CFD (computational fluid dynamics) simulations have become popular to predict airflow conditions. Nevertheless, experimental evaluation is still inevitable. Especially for the case of porous media, there is little experimental verification for applications with complex geometries, e.g., a mixture of airflow in free space and porous media. The optimization of airflow in cooling applications for food products is an example with high economic importance, e.g., the cooling of agricultural produce in cold stores. 

Rapid cooling of fresh horticultural produce after harvest is important to avoid quality loss before offering it to consumers. In forced-air precooling facilities and cool stores, the field and respiration heat of the produce is removed by circulation of cooled air. During initial cooling processes after harvest, produce is ventilated with higher flow rates compared to the following storage period when the set point temperature is achieved. The air speed and the temperature of the airflow affect not only the cooling rate of the produce but also their water status. High airflow increases undesirable water loss due to transpiration at the produce surface. This effect is especially important for fruit and vegetables with high surface permeability to water vapor [1,2]. Uniform air speed and temperature distribution in a produce stack are required to maintain homogenous quality. There is an increasing research interest in analyzing airflow conditions, heat and mass transfer in cooling facilities to derive appropriate layout and operation of storage and transport facilities and package design [3,4,5,6]. Flow conditions are very complex due to high variability of produce shapes, package geometry and arrangement of packages in transport and storage facilities. Airflow measurement techniques were applied according to specific conditions in cooling facilities or packages with only few verifications of the airflow inside porous media, i.e., between fruits.

To characterize airflow, the terms air speed and air velocity have to be differentiated. “Speed” is a scalar quantity that describes the change of rate of distance in time of an object in contrast to “velocity” describing a vector quantity expressing both magnitude and directions [7]. The term “air speed” is used to characterize air movement with unknown direction. The most applied principle for measuring the speed or the velocity of air in industrial food cooling facilities is thermal anemometry. 

Air velocity was measured with directional hot wire anemometers in gaps between pallets or bins in fruit and vegetable cold stores. Different flow directions were considered by changing the orientation of the sensors [8,9]. Omni-directional hot film anemometers were used for air speed measurements in cold stores, because of the unknown flow direction at the measuring points in gaps between product bins and above the bin stacks [10,11,12]. Laguerre et al. [13] measured the air velocity with hot wire anemometers in the homogenized airflow in a supply air duct and in the air curtain of a display cabinet for cooling food packages in retail. Hot wire anemometers are not suitable for complex situations with high fluctuations [13] or airflow measurements inside packages or bulks because of the size of the probes up to 0.5 m length [14]. Alvarez and Flick [15] and Delele et al. [16] studied air velocity and turbulence distribution inside vented bins for agricultural products with hot wire anemometer measurements. For fixing the rod-type sensor probe between synthetic spheres as produce dummies in a bin, it was inevitable to build a gap by removing some of the spheres or using a cylindrical frame for the sensor to avoid contact to the spheres. Concentration changes of tracer gases in moving air were also used for velocity determination in the surrounding of horticultural produce. Tanner et al. [17] measured air velocity at different points in a ventilated package of horticultural products by analyzing the reduction of CO_2_-concentration at different positions inside the package compared to the CO_2_-concentration in the inlet stream. The air in the package was sampled with a pipe and conducted to a CO_2_ gas analyzer. This method is limited by the possibility to place the pipe and not suitable for continuous measurement. To investigate airflow profiles more accurate techniques are available as laser Doppler anemometry (LDA) and particle image velocimetry (PIV). These methods allow determination of air velocity in the free space by analyzing tracer particle displacement [14] at defined measuring points (LDA) or in a field (PIV). Moureh et al. [18] used LDA for measurement of airflow in a reduced-scale model of a truck for food transport. The air velocity and fluctuations were measured above the pallets. This measurement technique requires space for influx of tracer particles and measuring positions accessible for the laser light beam. Therefore, measuring points can be chosen only in gaps or above the products but not inside packages or bulks. Air velocity fields were determined with PIV in the air curtain in front of a display cabinet [13]. The measurement agreed well with measurements of a hot wire anemometer in zones of uncomplex flow conditions at the internal side of the curtain. In zones with rapid velocity fluctuations the punctual measurements of the hot wire anemometers showed discrepancies to the air velocity fields recorded by PIV. Ferruah et al. [19] studied velocity fields with PIV in different horizontal planes of a transparent model of a typical retail clamshell filled with synthetic spheres. Similar to the LDA method, the PIV technique captures air velocity only in space, which is accessible to laser light. A widely applied method for indirect estimation of airflow inside packages or produce stacks is the determination of the convective heat transfer coefficient (CHTC) with aluminum or brass heating spheres equipped with a thermocouple and surrounded by synthetic spheres to minimize the influence by conduction or radiation between the heating sphere and the surrounding area. The CHTC is calculated from the relation of time and temperature decrease of the sphere [8,18,20]. Studies with heated spheres for CHTC determination were carried out in produce stacks and in packages with regular arrangement of produce dummies in the surrounding of the measurement device.

The objective of this study was to develop a sensor for measuring air speed between horticultural produce in bulks and storage bins with random stacking of the products. It is envisaged that such measuring system would be applicable in industrial field tests as well as allow continuous measurement to record adaptation of air speed next to the produce with changing airflow conditions in cold stores. The air speed measurement should be omnidirectional as the airflow direction in the hollow space of a bulk is unknown and not relevant for the presented application. The measuring system itself should affect the speed values as minimal as possible. It should be able to record low air speed values below 1 m/s. To our knowledge, there is no system available for measurement of air speed distribution inside a bulk or a bin with random produce stacking. The estimation of local airflow by preheated spheres does not allow real time measurement and capture of air speed fluctuations. 

In this work, the construction and calibration of a new airflow sensing system is described. The new device was tested for airflow measurement between fruit dummies in a wind tunnel at laboratory scale and between fruit in an apple bin in a commercial storage room.

## 2. Structure of the Airflow Measuring Device

**Figure 1:**
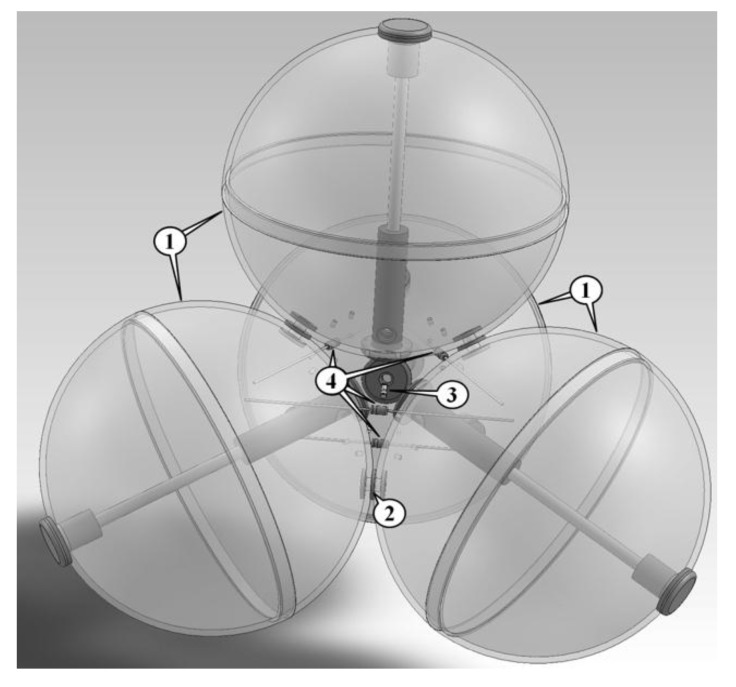
Schematic of the structure of the sensing device (1: polystyrene spheres; 2: screws; 3: central silicon diode; and 4: the four silicon diodes in the outer gaps).

The airflow speed logger (ASL) consists of four hollow transparent spheres made of polystyrene (thickness 1.5 mm). Each of the spheres has a diameter of 80 millimeters. Using screws, the spheres are mounted together in the form of a triangular pyramid similar to how they would arrange themselves when closely packed in a bulk of fruit with similar shapes and sizes (Figure 1).

**Figure 2:**
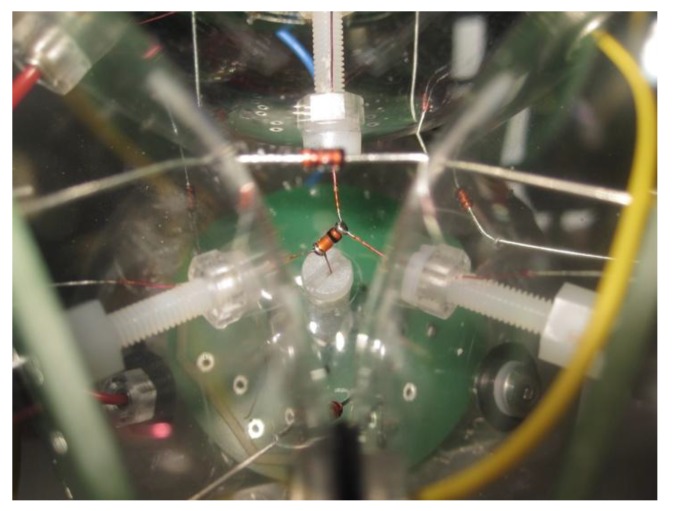
Detail of the central silicon diode fixed in the space between the four spheres of the sensing device.

At the central position in the space between the spheres, a silicon diode is attached by four wires. This diode is the main sensor element. Within the gaps between the three spheres of each of the four pyramidal faces, four further diodes are fixed with wires acting as reference diodes. These four diodes are connected electrically in series (Figure 1 and Figure 2).

**Figure 3:**
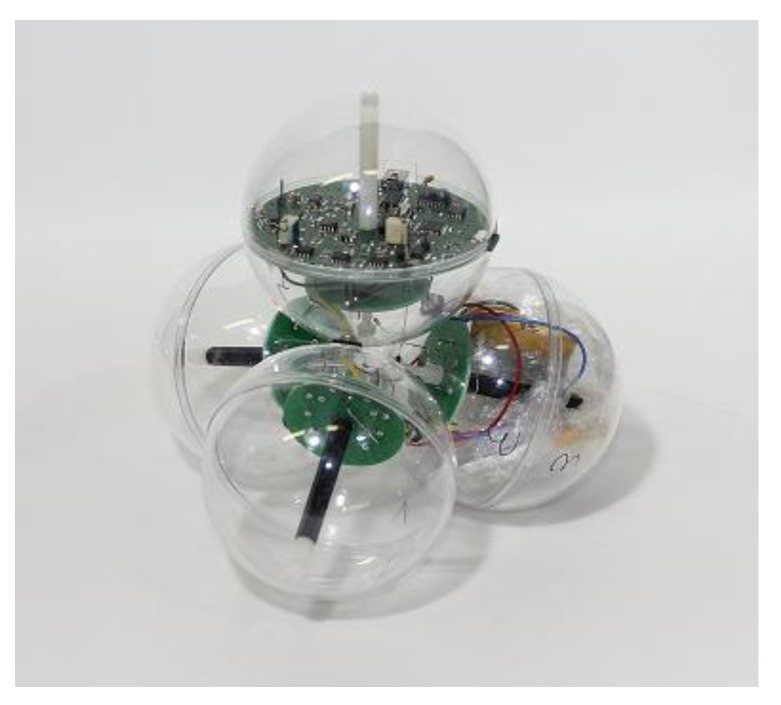
The completely assembled air speed measuring device.

**Figure 4:**
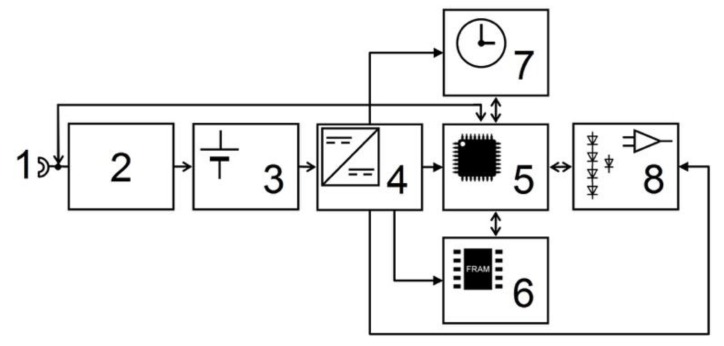
Schematic diagram of the electronic circuit (1: USB jack; 2: battery charger; 3: LiPo-battery; 4: DC-DC converter; 5: micro controller; 6: memory; 7: real time clock; and 8: analog circuitry).

Beside the diodes acting as airflow and temperature sensor elements, the self-contained device is equipped with electronic components that are necessary for running the measuring system. Two batteries are placed inside one of the four spheres. The electronic circuity is located in one of the adjacent spheres (Figure 3). A printed circuit board (PCB) contains an analog part (8), a microcontroller with A/D converter (5), a USB port (1) for data transfer, a non-volatile data memory (ferro electric RAM (FRAM)) (6), a real time clock chip (7), several DC-DC converters (4), and a charging circuitry (2) for the two lithium polymer batteries (3.3 V/900 mAh) (3) (Figure 4).

## 3. Measuring Principle 

The measurements are based on the calorimetric principle operating in CTD (Constant Temperature Difference) mode. This principle has the advantage of a nearly omnidirectional sensitivity for the airflow. Furthermore, the CTD mode causes the sensitivity to decrease while air speed increases resulting in a wide dynamic range. The measuring principle is based on cooling by the airflow of an electrically heated element (central diode) serving simultaneously as heater and as temperature sensor. The four unheated reference diodes, which are exposed to the airflow simultaneously, are used for determining the difference between the temperatures of the heated diode and ambience as needed for the CTD method. The central diode is heated by a controlled DC current and cooled by heat transfer loss caused by the airflow. Every 100 ms, the heating DC current is interrupted for 2 ms. During this period of 2 ms, small measuring current flows through the diode to measure the voltage drop across the diode chip which is linear proportional to the temperature. Measurement current also flows through the four reference diodes connected in series. The voltage drop across these diodes is linear proportional to the ambient temperature and can therefore be used to measure this temperature. 

**Figure 5:**
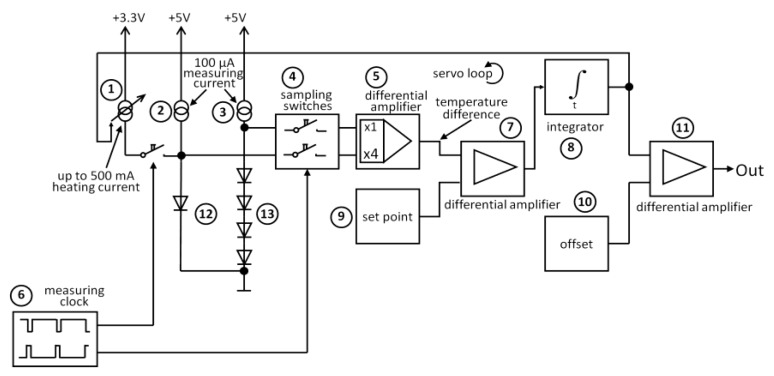
Simplified diagram of the analog loop controlled system for adjustment of the heating current (CTD mode) (representing Part 8 in Figure 3).

Figure 5 shows the simplified diagram of the analog loop-controlled system which is made to adjust the heating current (1) in such a way that the temperature difference between heated and unheated diodes remains at a constant level independent of the ambient temperature. Therefore, a measuring clock (6) is generated by the microcontroller to intermittently switch on and off the heating current (1) and to control the sampling switches (4) for the voltage measurement. Every 100 ms, the current is interrupted and measuring current flows through the heated diode (12) as well as the reference diodes (13). A differential amplifier amplifies the weighted voltage difference between the diodes. Its output voltage is proportional to the temperature difference between heated and unheated diodes. A second differential amplifier (7) compares this voltage difference with a preset voltage and an integrator (8) integrates the voltage variance to control the heating current source (1). A third differential amplifier (11) subtracts an offset (10) such that there is nearly zero voltage at the output when air speed is set to zero. The present heating current in the balanced loop is directly proportional to the airflow speed of the cooling air passing the central diode. The OUT signal (Figure 5) is led to the A/D converter input of the microcontroller, to be digitized and stored. The microcontroller is controlling the whole system. The data are stored (recorded) every second together with the ambient temperature data and the clock cycle. After completion of the measurements, the recorded data have to be transferred to a PC via the USB port. The lithium polymer batteries for supplying the measurement unit are also charged from the USB port. The capacity of the battery and the data memory is adequate for the sensing system to record continuously up to 28 h. The measurement may be interrupted up to 99 times along a variable period before continuation of operation.

## 4. Calibration of the Sensor

**Figure 6:**
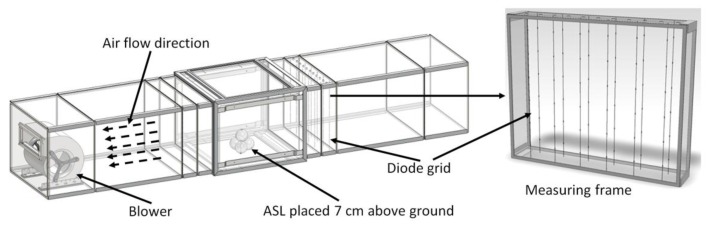
Wind tunnel with sensor placed for calibration and measuring frame with diode grid for average air speed measurement.

The calibration of the measuring device was performed in a laboratory sized wind tunnel of 3.3 m length and sectional area of 550 mm × 450 mm with walls made of acrylic glass. The airflow in the tunnel was generated by a continuously adjustable cross-flow blower (type D340/E1, Fischbach, Luft- und Ventilatorentechnik GmbH, Neunkirchen, Germany). The ASL was placed centric between the side walls on a platform at a height of 7 cm above the ground of the tunnel (Figure 6).

**Figure 7:**
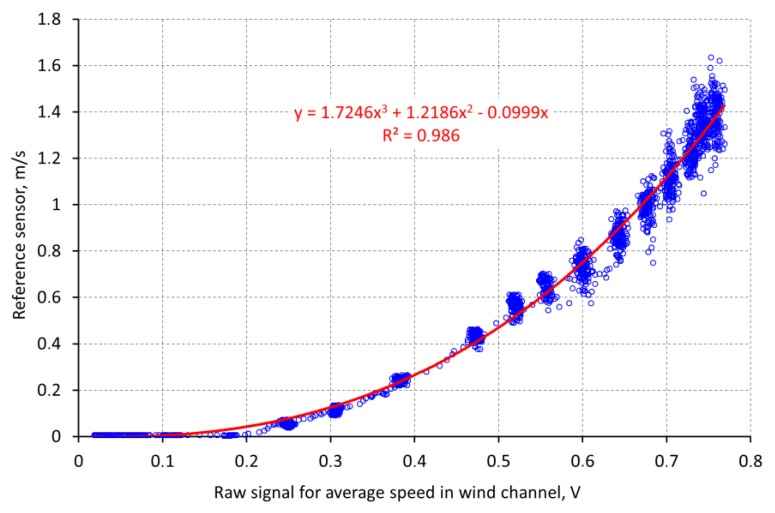
Local air speed (reference sensor) at the central diode as a function of raw data recorded by the diode grid.

The aim of the calibration was to calibrate the ASL to the air speed present at the location of the heated diode in the center of the ASL, which is equivalent to the gap between apples arranged similar to the plastic spheres. Therefore, the reference sensor used for the calibration was placed temporarily at the same spot. Neither the geometry of the wind tunnel nor the orientation of the ASL could influence the result of the calibration as long as it was possible to repeatedly reach a time invariant airflow at this spot. A thermal anemometer (Velocity transducer model 8465, TSI Inc., St. Paul, MN, USA, accuracy ±0.5% of full scale of selected range) was used as reference sensor with a measuring range of 0–5 m/s. This special device has the advantage of using a very small sensor probe that can simply be placed very near to the diode of the ASL. The ASL and the reference sensor are working according to the calorimetric principle. Therefore, both the diode of the ASL as well as the sensor head of the thermal anemometer are sensitive to local heating sources. To avoid interferences between both sensors acting at nearly the same location, they had to be used consecutively. First a set of increasing air speed steps was measured with the reference sensor, while the ASL was switched off. In a second part, the ASL had to record raw data in the same range of air speed data while the reference sensor was switched off (Figure 7). Airflow generation is controlled only by changing the voltage of the blower. An additional sensor was needed to ensure that the air speed in the wind tunnel during the recording of the reference data is comparable to the air speed present while recording the data with the ASL. A grid of 2 × 16 diodes interconnected with thin wires was mounted at a measuring frame in the tunnel (Figure 6). This grid of diodes was used for monitoring the average air speed within the cross-section of the tunnel by means of the same measuring principle as for the ASL. As a result of the first calibration part a correlation function was calculated for the relation between the raw data of the diode grid and the real air speed locally measured with the reference sensor (Figure 7).

Since the reference sensor does not measure air speed values below 0.1 m/s, the range from 0 to 0.1 m/s is extrapolated using the function that was fitted to the measured data. It was done in that way, because the data above 0.1 m/s are reliable and the point of origin is known. We can assume that the calibration function is a steady one without any discontinuities.

**Figure 8:**
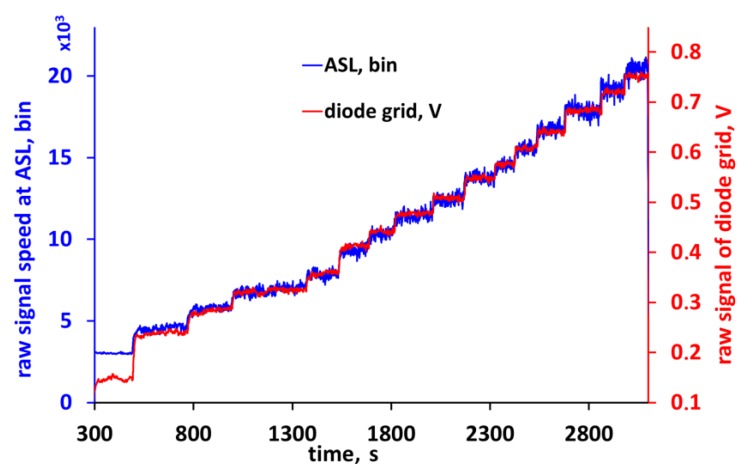
Raw data recorded by the ASL and by the diode grid.

During the second part of the calibration procedure the raw data of the diode grid were recorded (Figure 8). Due to the nonlinearity of the diode grid at very low air speed, there is a difference at the first step. Since the diode grid is used in both parts of the calibration, this effect does not affect the result. Using the function in Figure 7, in a next step, the raw data of the diode grid were converted into real air speed data at the location of the diode in the ASL.

**Figure 9:**
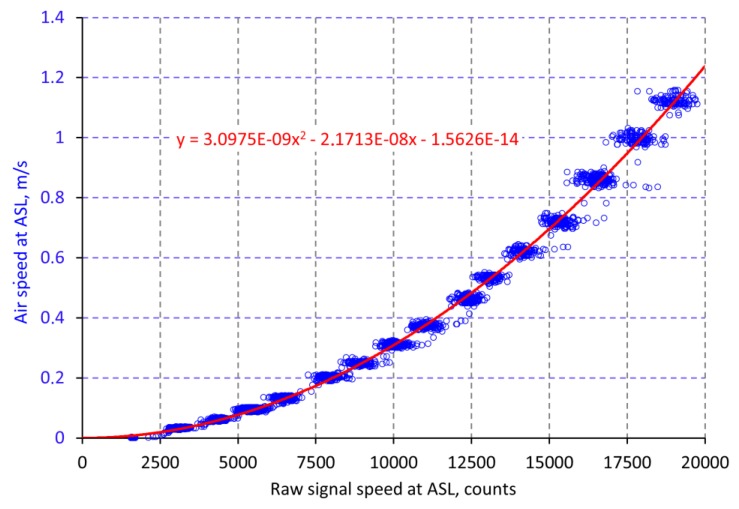
Relation between the raw signal (binary data) of the sensing device ASL and the air speed measured by the reference sensor (calibration function).

The final result was the relation between this real air speed data measured by the reference sensor and the raw data recorded by the ASL (Figure 9). This function is used to calculate the real air speed from the binary data of the A/D converter of the ASL.

**Figure 10:**
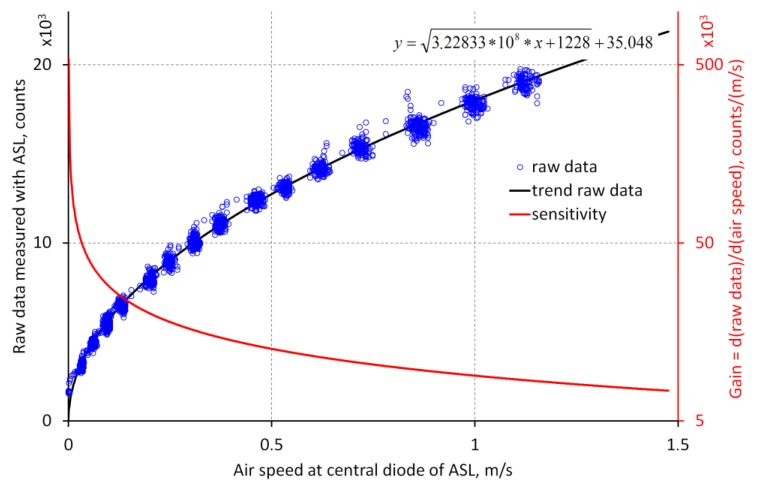
Sensitivity and gain of the ASL.

The inverse function (Figure 10) of the function in Figure 9 represents the sensitivity of the device. The derivative of the sensitivity is the gain of the sensor and tells how many binary steps will be present by a given change in air speed. It decreases from about 500 binary steps for 1 mm/s at an air speed near 0 mm/s (resolution of 2 µm/s) to about 7 binary steps for 1 mm/s at 1.5 m/s (resolution of 143 µm/s). This diagram proves the high dynamic range of the sensor which is limited especially by the stability of the zero point.

The peak-to-peak noise at zero air speed—while ALS was placed in a closed chamber—was about 0.5 mm/s. Further investigation relating the accuracy of the ASL will be performed if a small reference sensor is available for lower air speed that fits in the space between the spheres. Changes of the ambient temperature can influence the result, as the measuring principle bases on the constant temperature difference of two diode items. Software compensation ensures that the zero level of the ASL keeps stable (±2 mm/s at 20 K temperature difference). Especially fast changes of the temperature cause the effect that the parts of the ASL have temperature different to the air. Therefore, the diodes are influenced by the temperature of the airflow as well as by that of the device itself. Such temperature changes can temporarily shift the zero level. For a step of 20 K (cooling from 24 °C to 4 °C), the zero level was shifted for about 5 min to a maximum difference of 10 mm/s. Since the temperature is recorded together with the air speed, such effects can be considered when evaluating the results.

## 5. Application of ASL for Air Speed Measurements in Produce Bulks

### 5.1. Measurement on Laboratory Scale 

**Figure 11:**
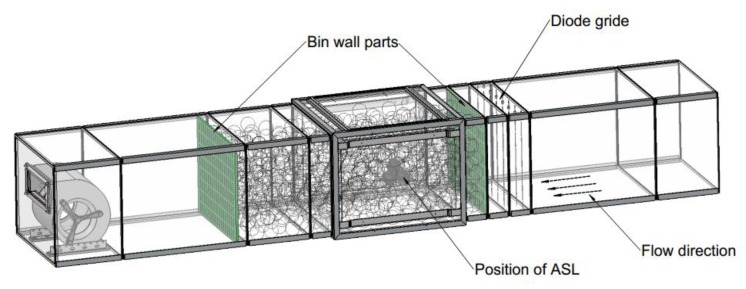
Wind tunnel filled with plastic spheres.

To measure air speed inside a bulk, the same laboratory wind tunnel as for the sensor calibration (Figure 6) was used. The wind tunnel was filled randomly with plastic spheres (Ø 70 mm) in a section of 1.15 m length between the diode grid and the blower (Figure 11). To simulate practical conditions, two wall parts with typical openings of a commercial apple bin were placed in front and behind the bulk. The air speed measuring device was placed at the center of the bulk between the plastic spheres. Airflow was directed through the bulk by increasing stepwise the speed of the upstream airflow in the range of 0.1 to 0.8 m/s in intervals of 15 min measuring time. The measurement was done at ambient temperature of 23 °C.

**Figure 12:**
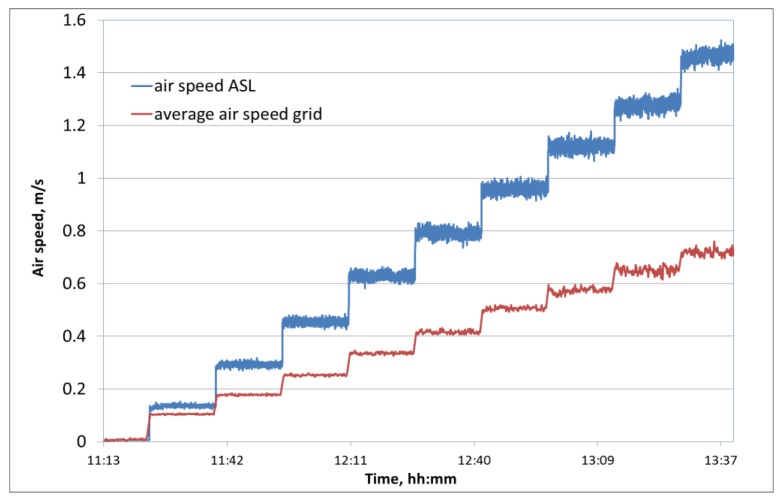
Simultaneous measurement of the air speed in the upstreaming airflow (grid) and in the bulk (ASL) inside the wind tunnel.

Figure 12 presents the non-directional air speed measured by ASL with a scanning rate of 1 s. In the gap between the spheres, air speed deviations were measured, which were highly stable during the measurement period of 15 min at each level of upstream airflow. The measured air speed deviations occur due to flow turbulences as it was shown for airflow through a stack of spherical produce in a bin [3,15]. With increasing flow speed, the standard deviation from the average airspeed, increased from 0.005 m/s at low average speed of 0.14 m/s to 0.02 m/s at average flow of 1.46 m/s. Test measurements of the device in a surrounding without airflow inside a closed plastic bag showed that the random noise of the sensor system itself is negligible with a standard deviation of 1.28 × 10^−5^ m/s at an average zero point of 4.4 × 10^−5^ m/s.

The air speed at the exemplary measuring position of the ASL in the bulk of produce dummies was higher than the incoming average airflow (measured by the grid) due to the reduction of the cross section inside the bulk compared to the area in front of the bulk. Other than in storage applications, the airflow could not bypass the fruit bulk through gaps between storage boxes. This effect was also shown by Delele et al. [16] by simulating airflow through a vented box with randomly stacked spherical products. The ratio between the measured speed at the diode grid and the ASL is related to the ratio between superficial (u_s_) and true speed (u) in the bulk, which can be calculated by u_s_ = φ u where φ = 0.4 is the porosity of the apple bulk. By calculating the true speed in the bulk from measured values of the ASL, the difference between the calculated true speed and the measured grid speed is lower than 20% independent on the level (standard deviation of difference 3.6%). This difference between calculated and measured speed depends on orientation and position of the real pore in the bulk. Hence, it could be shown that the sensor is suitable to measure the true speed in a pore in the bulk.

### 5.2. Application of ASL in an Apple Bin in a Cold Store

**Figure 13:**
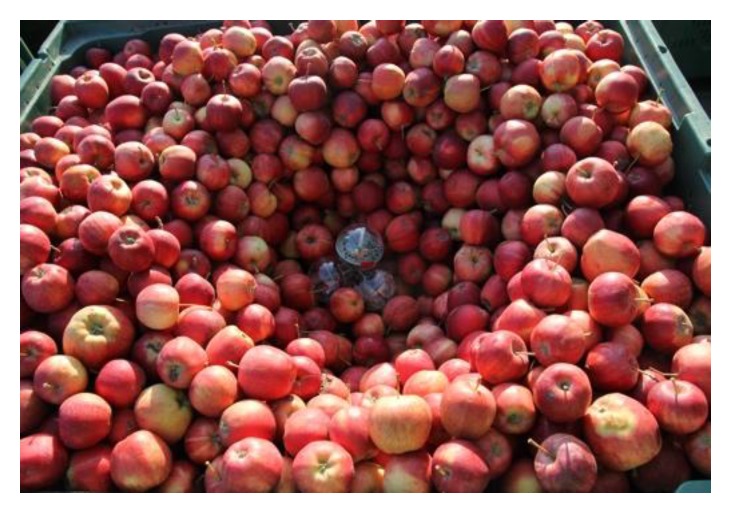
Position of the device for airspeed measurement between apples in a storage bin.

The application of the ASL (air speed and temperature recording) was tested in a common industrial apple cold store (340 m^3^) stowed with 216 plastic bins, 8 bins high. The device was placed inside one bin filled with 300 kg apples at the center of the fruit bulk and covered with a fruit layer of about 20 cm height (Figure 13). The bin was placed in one row of bin stacks near the room wall at a central position in the stack with neighboring bins above and beside the bin fitted with the device.

**Figure 14:**
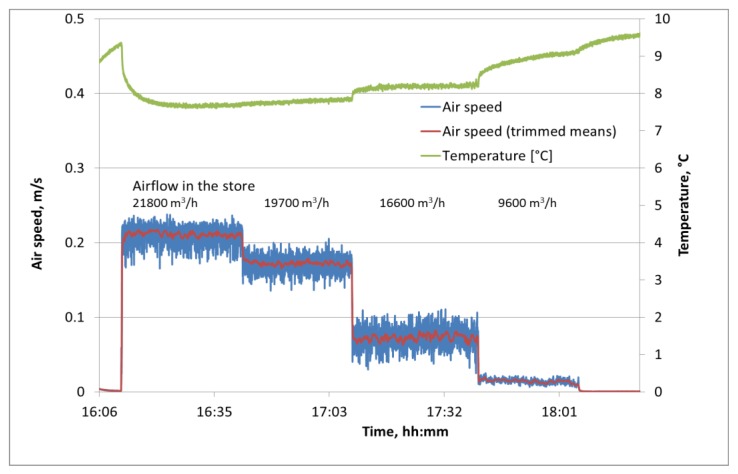
Air speed and temperature measurement with ASL in an industrial apple storage room (trimmed means: maximum and minimum values are eliminated).

Four fans were situated at one side wall of the store above the bin stack for circulating air in the room without operation of the cooling unit. Fast temperature deviations during airflow measurement with a calorimetric system should be avoided because they might influence air speed measurement. The airflow of 21,800 m^3^/h volume was reduced stepwise to 9600 m^3^/h by decreasing the revolution speed of the fans after a measuring time of 25 min. at each of 4 levels. The air speed values measured by the ASL in the apple bin with unknown upstream air speed are in a much lower range than in the wind tunnel with forced airflow through the fruit bulk. The initial average air speed between the fruit decreased immediately after each reduction of the fan revolution in the store from 0.21 m/s (21,800 m^3^/h) to 0.01 m/s (9600 m^3^/h) (Figure 14). Similar to the measurements in the laboratory wind tunnel, the air speed between the apples in the bin fluctuated with decreasing standard deviation when the average air speed was reduced (e.g., 0.01 m/s at average of 0.21 m/s and 0.007 m/s at 0.01 m/s). The air speed values showed high stability at different levels of fan revolution despite slight temperature changes during the measurement period.

## 6. Conclusions

A new autonomous sensing device is developed for determination of air speed and temperature inside bulks and bins of stored agricultural products. Up to now, it was impossible to perform continuous airflow measurements in bulks of fruit or vegetables without disturbing the storage process. The presented device is adapted to the size of apples. It is appropriate in particular for produce of similar size and shape such as onions, potatoes, kiwi and oranges. The measuring principle is based on an established calorimetric method for air speed determination. The peculiarity of the device is the measurement of non-directional flow speed in gaps next to the produce due to its pyramidal array of four spheres simulating stacked fruit in storage conditions with central position of the heated diodes as main sensor element between the spheres.

The device meets the requirement of recording very low air speed in a range of 0 to 1.5 m/s, as is estimated between fruit in ventilated storage bins. The need for air speed measurement in a very low measuring range (<0.3 m/s) is exemplary shown by measurements in an industrial apple store. Therefore, an advantage is that the sensitivity of the sensor increases remarkably with decreasing air speed values. Further studies on the measurements with the device in produce bulks may be used for validation of air speed models. The new system allows measurements with much lower influence on the airflow in the produce stack by the sensing system itself than common hot wire anemometers that were used in previous studies. 

The handling of the device in industrial conditions is easy because it is placed without cable connection at the measuring position. The arrangement is relatively insensitive to mechanical damage compared to anemometers because the four spheres protect the sensor unit. The internal power supply and data memory allow data recording up to 28 h. The readout of the data and the battery charging is done with the same USB-connection. For application in storage rooms, the influence of fast temperature changes on air speed measurement must be considered and measurements should be done when temperature is stable. A correction factor could be established because of the simultaneous temperature measurement with air speed recording.

The new sensor will provide the possibility to better understand airflow conditions near produce in cool stores with the main task to improve quality of fruit, vegetables and potatoes.

## References

[1] Gaffney J.J., Baird C.D., Chau K.V. (1985). Influence of Airflow Rate, Respiration, Evaporative Cooling, and Other Factors Affecting Weight Loss Calculations for Fruits and Vegetables. ASHRAE Trans..

[2] Ben-Yehoshua S., Rodov V., Bartz J.A., Brecht J.K. (2003). Transpiration and Water Stress. Postharvest Physiology and Pathology of Vegetables.

[3] Verboven P., Flick D., Nicolai B.M., Alvarez G. (2006). Modelling transport phenomena in refrigerated food bulks, packages and stacks: Basics and advances. Int. J. Refrig..

[4] Dehghannya J., Ngadi M., Vigneault C. (2010). Mathematical Modeling Procedures for Airflow, Heat and Mass Transfer during Forced Convection Cooling of Produce: A Review. Food Eng. Rev..

[5] Ambaw A., Delele M.A., Defraeye T., Ho Q.T., Opara L.U., Nicolai B.M., Verboven P. (2013). The use of CFD to characterize and design post-harvest storage facilities: Past, present and future. Comput. Electron. Agric..

[6] Getahun S., Ambaw A., Delele M.A., Meyer C.J., Opara U.L. (2017). Analysis of airflow and heat transfer inside fruit packed refrigerated shipping container: Part I—Model development and validation. J. Food Eng..

[7] Walker P.M.B. (1995). The Wordsworth Dictionary of Science and Technology.

[8] Duret S., Hoang H.M., Flick D., Laguerre O. (2014). Experimental characterization of airflow, heat and mass transfer in a cold room filled with food products. Int. J. Refrig..

[9] Scaar H., Praeger U., Gottschalk K., Jedermann R., Geyer M. (2017). Experimental and numerical analysis of airflow in fruit and vegetable cold stores. Landtech. Agric. Eng..

[10] Hoang M.L., Verboven P., De Baerdemaeker J., Nicolai B.M. (2000). Analysis of the air flow in a cold store by means of computational fluid dynamics. Int. J. Refrig..

[11] Nahor H.B., Hoang M.L., Verboven P., Baelmans M., Nicolai B.M. (2005). CFD model of the airflow, heat and mass transfer in cool stores. Int. J. Refrig..

[12] Delele M.A., Schenk A., Tijskens E., Ramon H., Nicolai B.M., Verboven P. (2009). Optimization of the humidification of cold stores by pressurized water atomizers based on a multiscale CFD model. J. Food Eng..

[13] Laguerre O., Hoang M.H., Osswald V., Flick D. (2012). Experimental study of heat transfer and air flow in a refrigerated display cabinet. J. Food Eng..

[14] O’Sullivan J., Ferrua M., Love R., Verboven P., Nicolai B., East A. (2014). Airflow measurement techniques for the improvement of forced-air cooling, refrigeration and drying operations. J. Food Eng..

[15] Alvarez G., Flick D. (1999). Analysis of heterogeneous cooling of agricultural products inside bins Part I: Aerodynamic study. J. Food Eng..

[16] Delele M.A., Tijskens E., Atalay Y.T., Ho Q.T., Ramon H., Nicolaï B.M., Verboven P. (2008). Combined discrete element and CFD modelling of airflow through random stacking of horticultural products in vented boxes. J. Food Eng..

[17] Tanner D.J., Cleland A.C., Robertson T.R., Opara L.U. (2000). Use of carbon dioxide as a tracer gas for determining in-package airflow distribution. J. Agric. Eng. Res..

[18] Moureh J., Tapsoba S., Derens E., Flick D. (2009). Air velocity characteristics within vented pallets loaded in a refrigerated vehicle with and without air ducts. Int. J. Refrig..

[19] Ferrua M.J., Singh R.P. (2009). Modeling the forced-air cooling process of fresh strawberry packages, Part II: Experimental validation of the flow model. Int. J. Refrig..

[20] Alvarez G., Flick D. (2007). Modelling turbulent flow and heat transfer using macro-porous media approach used to predict cooling kinetics of stack of food products. J. Food Eng..

